# Role of Education and Mentorship in Entrepreneurial Behavior: Mediating Role of Self-Efficacy

**DOI:** 10.3389/fpsyg.2021.775227

**Published:** 2021-11-30

**Authors:** Binwu Hu, Qiang Zheng, Jie Wu, Zhibin Tang, Jianchun Zhu, Simin Wu, Ying Ling

**Affiliations:** ^1^Zhejiang University of Technology, Hangzhou, China; ^2^Hunan Normal University, Changsha, China; ^3^Jinhua Radio and Television University, Jinhua, China; ^4^Zhejiang Industry Polytechnic College, Shaoxing, China

**Keywords:** education, training, entrepreneurial self-efficacy, entrepreneurial behavior, intrinsic motivation

## Abstract

Farmers have been very precious for societies for ages. Their active experiments, valuable knowledge about their surroundings, environment, and crops’ requirements have been a vital part of society. However, the psychological perspectives have been a hole in the loop of farming. Hence, this study has investigated the antecedents of entrepreneurial behaviors of farmers with the mediating risk of their entrepreneurial self-efficacy (ESE). The population chosen for this study was the farming community of suburbs of China, and a sample size of 300 was selected for the data collection. This is a survey study, where a structured questionnaire was adapted on a five-point Likert scale. The data were collected from the farming community to know their psychological and behavioral preferences about their profession. This study has produced interesting results that education, training, and intrinsic motivation play a vital role in farmers’ ESE, affecting their entrepreneurial behaviors. This study will add to the body of knowledge and provide an eminent path for emerging entrepreneurs to find more mentorship opportunities to overcome the limitations in upcoming endeavors influencing education and training.

## Introduction

Numerous research on farmers’ education and entrepreneurial behavior revealed that there is a link between education and agricultural innovation (Yoshida et al., 2019). The link between education and entrepreneurship is a subject of conflicting data, and it might be positive or negative, significant or insignificant (Lipset, 2018). Farmers with a basic education were 8.7% more innovative and productive than farmers with no education, according to a World Bank survey performed in 1992 to assess the link between farmer education and agricultural efficiency in low-income nations (Bachewe et al., 2018). According to the World Bank’s findings, there is a favorable link between a farmer’s educational level and innovation in production (Zulfiqar and Thapa, 2018).

In a study on the impact of education on agriculture performed in Nepal, researchers discovered that education increases innovative agricultural production largely through boosting farmers’ decision-making abilities and, secondarily, by reducing their technical efficiency (Paudel et al., 2020). The phrase “technical efficiency” refers to a farmer’s capacity to make better input decisions and make more economically sound judgments. Entrepreneurship is a subject of study that continuously expands its boundaries to better comprehend it, including the farming sector (Dias et al., 2019). Some regard entrepreneurship as a distinct profession, similar to Schumpeter’s creativity as a vital engine of economic growth and employment creation. This is also the most prevalent reason why professionals and academics advocate for entrepreneurial education. Business historians pioneered the study of entrepreneurship between 1940 and 1950 (Pérez, 2019).

Nevertheless, the study of entrepreneurship came across severe methodological hurdles, leaving the research fragmented and marginalized. Globally, there has been a growing interest in entrepreneurship in recent decades. Entrepreneurship is now widely regarded as a source of job creation and economic growth (Kim et al., 2018). It is credited for beginning technical advancement, which is a key engine of socioeconomic progress. Entrepreneurship has the potential to open up agricultural prospects in China and drive growth in the economy (Qobo and Le Pere, 2018). The most significant aspect of a person’s entrepreneurial performance is his/her entrepreneurial intention. Studies have highlighted family education, economic growth, governmental, entrepreneurial orientation, and associated incentive programs and technical assistance, and geographical entrepreneurial atmosphere as essential determinants inside the entrepreneurial environment.

Furthermore, various psychological models of entrepreneurship have been presented to explain an individual’s entrepreneurial purpose and actions in light of the interplay between internal and external influences (Wang et al., 2016). The Theory of Planned Behavior is now the most important of these frameworks. Training can help you to develop the personality qualities, abilities, and skills needed and become an entrepreneur (An et al., 2021). Studies have found adverse association between financial performance and boardroom gender diversity (Ajaz et al., 2020). Earnings management plays a moderating role in the cash holdings (Sarfraz et al., 2020a).

Entrepreneurship is necessary for smallholder farmers’ survival in an ever-changing and increasingly complicated global market. Researchers say there are chances for expanding knowledge of the historical impact of values and culture on entrepreneurial behavior, using more careful techniques than in the past, and attempting to clarify the relevance of culture and its relationship to certain other variables (Xialong et al., 2021). There are several perspectives on who qualifies as an entrepreneur. Even though academics believe that a collection of entrepreneurial activities defines an entrepreneur, this set is not well defined. The goal of this desk research was to uncover farmers’ entrepreneurial habits. This study aimed to find an answer to the following research question: What characteristics of entrepreneurial conduct characterize farmers?

The farmers mostly acquire business abilities through a process of studying by doing rather than through formal schooling. Entrepreneurial education has been suggested as a necessary component of acquiring entrepreneurship and company management abilities (Šūmane et al., 2018; Shah et al., 2020). Entrepreneurial learning identifies and takes advantage of possibilities by starting, organizing, and managing a business socially and behaviorally. Entrepreneurship among the farming community contributes to multifaceted development in various ways, including assembling and harnessing various inputs, taking risks, innovating and imitating production techniques to reduce costs while increasing amount and quality, open marketplace frontiers, and organizing the production plant at different levels.

Starting a private enterprise like a farm may be difficult and time-consuming, so there is an increasing wealth of data on the entrepreneurial skills required to operate and expand a farm (Van der Burg et al., 2019). Mentorship can help farmers develop entrepreneurial skills. However, the effects of mentoring on entrepreneurial learning have only been studied to a limited extent (Ferreira et al., 2020). As a result, farmer-mentoring programs targeted at helping farmers’ development and learning have been examined to see how the mentoring idea is included, what types of learning are encouraged, and what impacts on entrepreneurship training are discovered (Permadi et al., 2020). The sociological dimension to entrepreneurial orientation involves engaging with other people, businesses, and those outside the company. The behavioral aspect of entrepreneurial orientation reflects the learning in both the farmer’s and the farming conduct (Niewolny and Whitter-Cummings, 2020).

Different types of farmer mentorship programs have been established to help farmers develop their entrepreneurial and farm management abilities, based on concepts from small business-supporting systems in non-agricultural industries (Sinyolo and Mudhara, 2018). While several studies discuss farmer mentorship programs, there are not many. The previous work focuses on discussing how these programs are put up. It is not specific about the benefits and drawbacks of such initiatives. Overall, studies of the impact of mentorship programs on entrepreneurship training have been undertaken (Jamaluddin et al., 2019). This is where the article hopes to help. As a result, we look into the benefits of entrepreneurial orientation from two previous mentorship programs. These programs assist farmers in honing their business and farm management abilities and putting them to good use.

The learning environment has a significant influence on self-efficacy views. Learning takes place in a social setting. The behaviors of others in the social environment and the intrinsic qualities of the culture in which learning occurs influence everyone’s constructs (Seah, 2018). Self-efficacy aids learning by encouraging endurance and giving the impression that one can attempt new approaches. As farmers grow more efficient, they become more conscious of how their new information is built on top of their prior knowledge. Agricultural extension education programs, for example, can offer farmers new information to boost self-efficacy while engaging in vicarious, enactive, and social experiences (Widyani et al., 2017). While there is a wealth of literature on educating and mentoring farmers to improve their entrepreneurial behavior, research on the use of self-efficacy as a mediating variable in farmer entrepreneurial behavior is still missing (Al-Shammari and Waleed, 2018). Limited research has utilized self-efficacy as a mediating variable in farmer entrepreneurial activity, according to this study.

According to literature, research using self-efficacy as a mediating variable has been conducted in academic motivation, career intention, organizational citizenship behavior, and treatment adherence (Klassen and Klassen, 2018). Self-efficacy has been utilized as a mediating variable by certain investigations. However, they have focused on other criteria such as goals and achievement, ethical leadership, technical inventiveness in sports, and the perceived academic atmosphere. Furthermore, previous research emphasizes self-efficacy as a predictor of information sharing behavior. Because there is minimal research investigating the mediation impact of self-efficacy on the entrepreneurial behavior of farmers, particularly in the agricultural environment, self-efficacy is used as a mediating variable in this study. This study revolved around certain objectives as follows: (1) To identify the role of education and training to entrepreneurial self-efficacy (ESE), (2) To analyze the role of mentorship and intrinsic motivation to self-efficacy, and (3) To investigate the antecedents of entrepreneurial behaviors of farmers with the mediating risk of their ESE.

## Literature Review

### Education and Training on Self-Efficacy

Entrepreneurship is a skill that may be gained through education (Nowiński et al., 2019). Among the essential sources of economic progress is entrepreneurship (Wardana et al., 2020). Farmers have emerged as rising entrepreneurial subjects due to legislative incentives and the current economic circumstances (Peng et al., 2015). Entrepreneurship is widely viewed as a significant and successful means of addressing challenges such as agricultural development, farmer revenue, and the farming industry, and it has attracted public attention (Elnadi and Gheith, 2021). Studying the elements that influence their motivation to innovate might help entrepreneurs to improve their position and performance. This research examines the effects of farmers’ entrepreneurial education and self-efficacy on their entrepreneurial orientation from the framework of perceived behavioral control. Entrepreneurial education has a considerable favorable impact on farmers’ entrepreneurship intention but no apparent impact on their entrepreneurial intentions (Nurlaela et al., 2020). According to this research, entrepreneurship can be learned by “learning by doing” in the course about becoming an entrepreneur, as well as from related entrepreneurship courses. Entrepreneurial education strives to improve the quality of entrepreneurship, aspiration, drive, innovation, and entrepreneurial spirit among farmers in order to prepare them for a certain profession, organization, or business strategy (Karimi, 2019). It also attempts to help entrepreneurs acquire the conceptual resources and competencies they need to succeed and uncover and recognize business possibilities. Several entrepreneurial training programs have been hosted by universities and linked external institutions in recent years, and these programs have steadily received recognition. Farmers in such programs are typically aspiring business people or entrepreneurs who believe they will lack the necessary knowledge and skills after beginning a business (Abraham, 2020).

These participants hope that by participating in such programs, they will develop their entrepreneurial skills and gain the ability to generate, comprehend, and pursue possibilities. In social psychology, behavior is described as a personal perception that includes subjective evaluations of oneself, people, affairs, actions, and events, among other things. It also significantly impacts a person’s responses and conduct (Darmanto and Yuliari, 2018). Entrepreneurial education is said to instill a sense of entrepreneurship in people and influence their perception and motivation. Entrepreneurial training and education can increase people’s managerial skills while also changing their awareness and attitudes regarding entrepreneurship (Liu et al., 2019). The goal of entrepreneurship education is to assist people in developing their entrepreneurial skills. As a result, this hypothesis suggests that an individual’s attitude toward entrepreneurship is strongly connected to their business expertise. Self-learning and external entrepreneurial spirit training can strengthen the farmers’ understanding of the entrepreneurial process and infuse them with a proactive approach (Fuller et al., 2018).

When it comes to beginning a new firm, entrepreneurs believe that having a strong entrepreneurial intention is a must. Entrepreneurial purpose refers to a person’s determination to start a new business and to see it through to completion. Studies have shown that entrepreneurial training boosts entrepreneurs’ entrepreneurial intentions and behaviors and improves their entrepreneurial performance (Akhtar, 2021). As a result, we believe that entrepreneurial education can help farmers with entrepreneurial orientation or potential to build entrepreneurial skills and knowledge and boost their chances of launching a firm (Liang and Chen, 2020). Keeping in view the role of education for entrepreneurship, the following hypothesis was devised.

***H1*** *: Education and training play a role in ESE*

### Mentorship in Entrepreneurial Self-Efficacy

Entrepreneurial self-efficacy seems to be triggered in part by entrepreneur mentorship (St-Jean and Tremblay, 2020). Compared with someone with low self-efficacy, a farmer with high self-efficacy is more willing to pursue and complete a task (Elliott et al., 2020). The level of reported self-efficacy in one area is frequently unrelated to perceived self-efficacy in some other (Baluku et al., 2020). Scholars of entrepreneurship have established the concept of “entrepreneurial self-efficacy” to concentrate on activities in the entrepreneurial domain to improve the prediction performance of self-efficacy assessments (Neneh, 2020). According to social learning theory, the most crucial contributions to enhancing self-efficacy in the mentorship relationship are parallel learning and motivation from mentors. Although theoretically and empirically support the impact of mentoring on self-efficacy, particularly in the entrepreneurial context, longitudinal data illustrate this relationship (Dunning, 2021). As a result, whether mentorship has a long-term or short-term influence on self-efficacy, as well as the circumstances under which this effect might be sustained, are yet unknown (Akyavuz and Asici, 2020). The primary purpose of this research is to see if mentoring could help beginner entrepreneurs to build their ESE. Entrepreneurial mentorship matches a new entrepreneur with a seasoned one who can offer guidance and methods of thinking to help the newbie avoid expensive and even deadly blunders (Blaique and Pinnington, 2021; Pereyra et al., 2021). Government agencies have put initiatives to assist entrepreneurs in the early stages of their business; mentorship is one of these programs (Hillemane, 2020). Mentorship is a term that comes from Homer’s Odyssey, in which the hero Odysseus entrusts his son Telemachus to his companion Mentor while he is at war. A mentor is a person who, influenced by Greek mythology, has specific attributes or is in a place of authority and who compassionately watches over a younger person so that they might benefit from the mentor’s support and counsel. Mentoring assistance is provided in various settings, including, but not limited to, aiding impoverished adolescents (Jafar et al., 2021).

This research is about mentorship in a stand-alone aggregate capacity, face-to-face, structured process with benevolent, accomplished business professionals who want to give back to local communities by assisting beginner entrepreneurs (Kuratko et al., 2021). Mentors help mentees to develop self-efficacy by providing vicarious experiences as positive examples, allowing them to evaluate and enhance their entrepreneurial and business competencies through social comparison and imitation (van Esch et al., 2021). Mentorship functions evaluate the strength and depth of the mentorship received and so serve as a substitute for the relationship’s effectiveness (Lefebvre et al., 2020). We propose the following hypothesis, knowing that performing mentorship responsibilities throughout a mentee will likely increase the farmer’s self-efficacy. To analyze the role of mentorship toward farmers’ self-efficacy, the following hypothesis was formulated.

**H2**: *Mentorship plays a role in ESE*

### Role of Intrinsic Motivation in Entrepreneurial Self-Efficacy

The relevance of task variety and task identity includes job importance, freedom, feedback, and psychological states, including work purpose, experienced accountabilities, and awareness of work outcomes (Çetin and Aşkun, 2018). In this model, increasing task-related motivation necessitated numerous interventions, particularly at the organizational and managerial levels, while growing psychological states was partially dependent on the individual employee, as an experienced role for the outcomes and understanding of work results were also dependent on task complexity, layout, and managerial behaviors (Lazzara et al., 2021). The importance of personal characteristics reminded us of the potential impact of self-efficacy, which may manifest as increased responsibility for consequences and understanding of outcomes. In the association between core personality and in-role work performance, intrinsic motivation played a partly mediation role (Kelley et al., 2020).

The researchers also stressed the need to conduct this sort of research in a non-Western setting because few studies exist in this field. While attempting to anticipate the impacts of self-efficacy and daily job creation on work productivity, a mediator function for work satisfaction was identified (Miraglia et al., 2017). It is worth noting that intrinsic motivation differentiates from job enjoyment in that it is the result of an activity rather than the process of doing it. Intrinsic motivation arises as a result of engaging in a particular activity. On the other hand, work happiness usually refers to a state of flow (Fischer et al., 2019). We felt very confident in suggesting that intrinsic motivation would perhaps serve as an intermediary between self-efficacy and inspiration and between self-efficacy and achievement, with significant explanatory significant contribution from social cognitive theory (SCT), self-determination theory (SDT), and core self-evaluations theory. Similarly, we felt confident in suggesting that intrinsic motivation would perhaps serve as a mediator among consciousness and effectiveness, with job characteristics and accurate information (Mahasneh and Alwan, 2018).

**H3**: *Intrinsic motivation plays a role in ESE*

### Role of Entrepreneurial Self-Efficacy Toward Entrepreneurial Behavior

Entrepreneurship has been shown to significantly affect economic growth, creating jobs, and creativity in a country (Li et al., 2020). Entrepreneurial passion is linked to good thoughts and attitudes toward activities that are important to one’s self-identity. Self-efficacy is a basic element of SCT, which promotes farmers’ tendency to fulfill their obligations and meet their goals (Shaheen and AL-Haddad, 2018). When adjusted to a shared activity context, self-efficacy is regarded to become a very perspective-specific attribute that leads to a greater outcome-forecasting rate. The ability to establish creative business solutions and a higher level of entrepreneurial passion appears to be the basis of having entrepreneurial aspirations (Jiang et al., 2017). The environmental quality has been improved during coronavirus disease (COVID-19) (Sarfraz et al., 2020b). However, there is a resemblance between self-efficacy and expectation theory since both are personality tools. The latter would be cognitively founded on the following presumptions: the probability that exertion will lead to quality level and the possibility that competence will result (Shaheen and AL-Haddad, 2018). On the other hand, self-efficacy is engaged with implementing the activity rather than the consequence (Haddad and Taleb, 2016). It was shown that self-efficacy positively mediated the relationship between improvisation behavior and the entrepreneurial process. It would further underline how important it is to effectiveness and entrepreneurial behavior (Khalil et al., 2021).

The entrepreneurial choice is motivated by the entrepreneurs’ skills, understanding, expertise, intelligence, learning, and behavioral intention. As stated previously in this research, intentions can lead to organizational innovation if they are properly implemented; furthermore, motivating factors, skills, and comprehension all influence entrepreneurship behavior (McGee and Peterson, 2019). Entrepreneurial behaviors were formerly thought of as discrete units of individual effort that can be identified by an audience and seem to have significance for that audience; however, according to this description, entrepreneurial behavior is carried out by the people who combine to form these organizations, not by organizations or teams (Haddad and Taleb, 2016). The literature provided the basis for the creation of the following hypothesis.

**H4**: *ESE plays a role in entrepreneurial behavior*

### Mediation of Entrepreneurial Self-Efficacy in Role of Education and Training in Entrepreneurial Behavior

Entrepreneurship can help promote global entrepreneurship and innovation, speed economic growth, close the wealth gap across regions, tackle employment, diversity, and poverty issues, and encourage the long-term success of businesses (Morozova et al., 2019). Entrepreneurship may improve economic performance, achieve market development, expand job opportunities, and maintain employment levels; hence, the amount of entrepreneurship in a country is critical (Hernández-Carrión et al., 2017). Entrepreneurial intention directs people’s attention, experience, and behaviors toward a certain entrepreneurial goal. ESE is a necessary condition for entrepreneurship ability (Asimakopoulos et al., 2019).

Entrepreneurial self-efficacy could be used to anticipate possible entrepreneurs’ ESE and conduct (Schmutzler et al., 2019). Individuals with strong entrepreneurial consciousness believe the world is full of chances. In contrast, those who have levels of ESE see the world through the lens of cost and danger. People with high ESE are better at seizing possibilities for achievement, can more accurately forecast the future, and have much more energy to spend on entrepreneurial tasks in the face of problems, risks, and uncertainty (Şahin et al., 2019).

It is proposed that significant others’ normative beliefs regarding entrepreneurship impart an inherent resourcefulness quality, which supports the interactive effects of subjective norms upon entrepreneurial aspirations, performance expectancy, and thus entrepreneurial ambitions (Sims and Chinta, 2019). Self-efficacy is a person’s belief in his/her capacity to do a set of tasks or activities successfully. Self-efficacy, strongly linked to deliberate action, impacts an individual’s views of a circumstance and how he/she adapts to it (Al-Ghazali and Afsar, 2021). The following hypothesis was formed to test the significance of the mediating role of ESE.

**H5**: *ESE mediates the role of education and training in entrepreneurial behavior*

### The Mediating Role of Entrepreneurial Self-Efficacy for Intrinsic Motivation in Entrepreneurial Behavior

Self-efficacy has long been thought to be a significant predictor of entrepreneurial intent. Roblek et al. (2020) defined self-efficacy as “a person’s experience in his or her capacity to complete a task.” It is a person’s belief in his/her ability to complete a task or overcome a difficult situation. ESE is the perception that talents may be applied to accomplish specific goals (Schmitt et al., 2018). ESE is significantly associated with entrepreneurial intention, according to previous studies. In entrepreneurial education, self-efficacy is frequently utilized to accurately predict entrepreneurial ambitions (Zeb et al., 2019). In entrepreneurial intention, self-efficacy is commonly utilized to predict entrepreneurial intents better and explain the complicated entrepreneurial behavior of bringing the latest entrepreneurs (Mouselli and Khalifa, 2017). Individuals determine their skills to execute the anticipated activity based on how strongly or adversely stimulated they feel about a specific task before beginning a business. Starting a business involves various hurdles and risks to entrepreneurs (Oparin et al., 2017). As a result, to start a new profitable business, individuals must have faith in themselves that they will be capable of overcoming the various problems that may arise and achieve their objectives with the abilities they possess. As a result, numerous behavioral models have been expanded and adjusted to have included self-efficacy as a significant driver of entrepreneurial desire.

Entrepreneurial self-efficacy was also proposed to moderate the influence of proactive behavior and innovation on entrepreneurial intention in the study (Son et al., 2018). Self-efficacy is a concept drawn from social learning theory that refers to a person’s belief during his/her capacity to complete a task. External factors, observational learning, and social modeling all influence ESE, acting as both facilitators and barriers; consequently, ESE focuses on the emotional structure that allows people to believe they are capable of performing various tasks and behaviors in a dynamic environment (Ahmed et al., 2020). As a result, those with a strong sense of personal are more likely to start a new business. As a result, we shall forecast this theory. Based upon the literature, the following hypothesis was devised.

**H6**: *ESE mediates the role of intrinsic motivation in entrepreneurial behavior*

**FIGURE 1:**
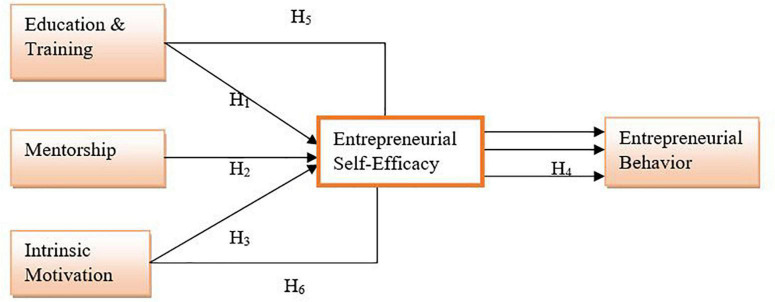
Conceptual model.

Based upon the literature review, this research was designed, and the following conceptual framework was developed. The research revolves around this, see Figure 1.

## Research Methods

In this section of the article, the methodology used in this study has been explained. The relationships for the hypotheses developed from the literature review are measured in this section. Variables of interest in this study are ESE and entrepreneurial behavior as a result. This study follows a post-positivist approach where the variables are quantified and measured using objective theories (Creswell and Creswell, 2018). Hence, among quantitative and qualitative approaches for data analysis, this study incorporates the quantitative methods for analyzing the data. The data is analyzed to obtain the results regarding the relationship between education and training, mentorship, and intrinsic motivation with entrepreneurial behavior and ESE risks involved (Mustafa et al., 2018). This is a cross-sectional study where the data was collected through a questionnaire designed with structured questions. The questionnaire was planned by adapting the scales used in previous studies for measuring the same variables. It contained 27 items in total following an interval scaling (Hernández-Carrión et al., 2017; Şahin et al., 2019; Al-Ghazali and Afsar, 2021). The population used in this study is the farmer community in China suburbs. The respondents were selected through convenient random sampling since approaching such a scattered community was a challenge in itself with time constraints. The total usable questionnaire in this study was 300. The data were analyzed using software SmartPLS 3.3.3. The demographic sheet used in this questionnaire contained five questions that included age, gender, education, and ownership or employed status with name as optional. The age and education were categorized into five brackets, while the status was categorized into two as owner or employed. The data obtained were analyzed using frequencies and percentages. The obtained results are mentioned in Table 1.

**TABLE 1:** Demographic summary.

**Demographic summary**	**Frequency**	**%**
**Gender**		
*Male*	238	79.33
*Female*	62	20.66
**Age**		
<*25*	24	0.08
*25–30*	18	0.06
31–40	67	22.33
*41–50*	105	35.00
*50*>	86	28.66
**Education**		
*Higher secondary*	169	56.33
*Bachelor*	98	32.66
*Masters*	21	0.07
*Doctorate*	–	–
*Others*	10	0.03
**Status**		
*Owner*	229	76.33
*Employed*	71	23.66

*N = 300.*

### Instrument Development

This study used a questionnaire that contained a demographic sheet and the structured items of each corresponding variable. The questionnaire consisted of 27 items; each item was measured with its particular scale developed in the past by different researchers. The scales were adapted accordingly. It was designed on a five-point Likert scale where the responses were classified into five categories ranging from strongly disagree to agree strongly. There were five variables in the questionnaire. The dependent variable of the study, that is, entrepreneurial behavior, was measured with eight items. The mediating variable, ESE, was measured with four items, while the independent variables were education and training with six items, mentorship with four, and intrinsic motivation with five items. The consolidated questionnaire was tested for reliability using Cronbach alpha (α) reliability and composite reliability. On the other hand, the validity of the data was checked with factor loadings and the correlations and heterotrait-monotrait (HTMT) ratio.

## Data Analysis

The data in this study were analyzed using the software SmartPLS 3.3.3. The statistical tool used for data analysis is structural equation modeling, measured in two stages in this software. The first phase of the analysis used the measurement analysis in which the data were checked for reliability and validity. This study has used the most practicing tests, that is, Cronbach alpha (α) reliability test and the composite reliability. The threshold for alpha (α) reliability, as mentioned by Hair et al. (2017), is 0.70. All the values in this study are above 0.70, ranging from 0.852 to 0.934 for alpha (α) reliability and 0.891 to 0.953 for composite reliability. Hence, the data in this study are reliable. As long as validity is concerned, the data are validated through factor loading. The threshold value for factor loading is said to be 0.60 (Nawaz et al., 2019, Nawaz et al., 2021). All the values in this study are above 0.60 except item M3, which is 0.50, acceptable (Hair et al., 2017). Moreover, the average variance extracted (AVE) should also be above 0.5 (Hair et al., 2017). Hence, the data showed convergent validity. These results can be seen in Table 2.

**TABLE 2:** Measurement model and descriptive statistics.

**Constructs**	**Code**	**FD**	**α**	**CR**	**AVE**
Education and training			0.871	0.891	0.577
	ET1	0.833			
	ET2	0.697			
	ET3	0.745			
	ET4	0.687			
	ET5	0.806			
	ET6	0.778			
Mentorship			0.934	0.953	0.836
	M1	0.898			
	M2	0.909			
	M3	0.897			
	M4	0.952			
Intrinsic motivation			0.852	0.897	0.645
	IM1	0.770			
	IM2	0.826			
	IM3	0.500			
	IM4	0.916			
	IM5	0.926			
Entrepreneurial self-efficacy			0.894	0.926	0.758
	ESE1	0.882			
	ESE2	0.850			
	ESE3	0.879			
	ESE4	0.872			
Entrepreneurial behavior			0.931	0.943	0.675
	EB1	0.860			
	EB2	0.829			
	EB3	0.832			
	EB4	0.815			
	EB5	0.836			
	EB6	0.787			
	EB7	0.802			
	EB8	0.808			

*CR, construct reliability; AVE, average variance extracted; α, Cronbach alpha.*

Additionally, the data were also convergently validated using the correlations *via* Fornell and Larcker criterion. The criterion for valid correlation results from this test is that the values in the diagonal, the top value in each column, is the highest than the rest of the values (Peterson and Kim, 2013; Hair et al., 2017). Hence, the data are valid in this study; see Table 3.

**TABLE 3:** Fornell and Larcker criterion.

Variables	E&T	EB	ESE	IM	M
E&T	0.760				
EB	0.597	0.821			
ESE	0.551	0.808	0.871		
IM	0.621	0.804	0.786	0.803	
M	0.468	0.206	0.267	0.375	0.914

*E&T, education and training; ESE, entrepreneurial self-efficacy; IM, intrinsic motivation; EB, entrepreneurial behavior.*

Another measure to check the validity of data is HTMT ratio. According to Hair et al. (2017), the cutoff value is 0.9. The results for this study meet this criterion; hence, making the data valid for use. The results can be seen in Table 4.

**TABLE 4:** Heterotrait-Monotrait ratio.

**Variables**	**E&T**	**EB**	**ESE**	**IM**	**M**
E&T					
EB	0.545				
ESE	0.518	0.880			
IM	0.681	0.882	0.883		
M	0.667	0.221	0.293	0.490	

*E&T, education and training; ESE, entrepreneurial self-efficacy; IM, intrinsic motivation; EB, entrepreneurial behavior.*

**FIGURE 2:**
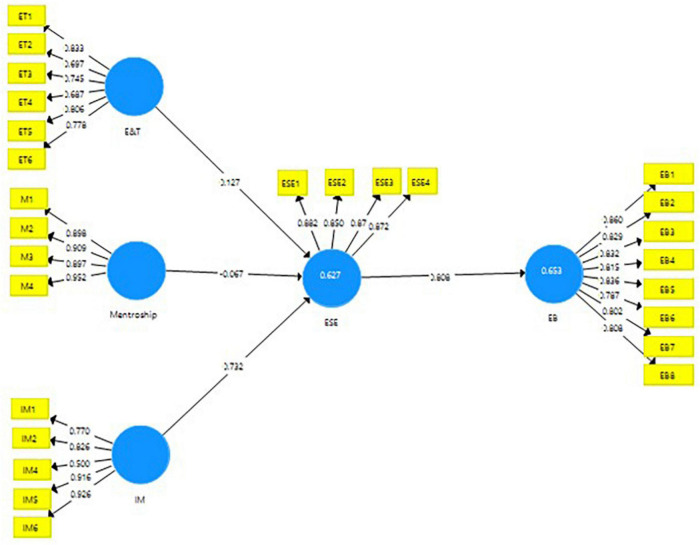
PLS-algorithm for measurement model.

**FIGURE 3:**
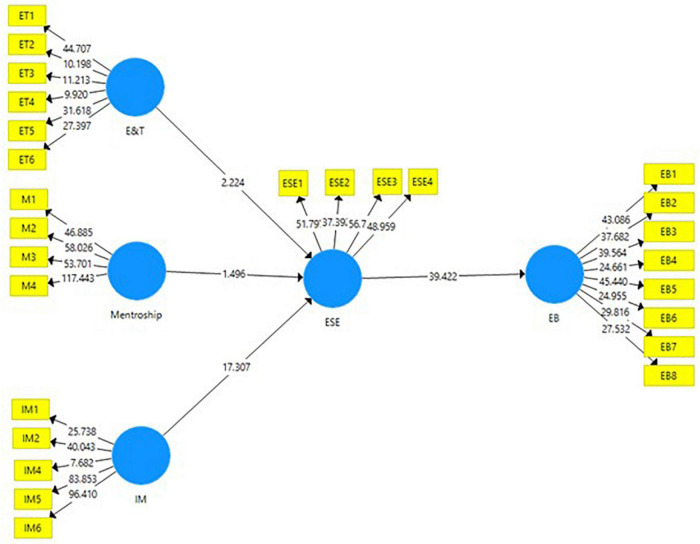
PLS-bootstrapping for structural model.

In the next phase of structural equation modeling through SmartPLS, the data are analyzed through a structural model *via* a consistent bootstrapping technique. In this stage, the linear relationships of the variables are measured. These relationships are shown in the form of path models. The straight lines show the direct effects, while the indirect effects are measured through the mediating variables. The results obtained can be seen in Figures 2, 3.

The results of the structural modeling are shown in the Table 5. There were six hypotheses in total. All hypotheses were supported in this study except for mentorship could not find any significance in predicting ESE (*t-statistic* = 2.387; *p-value* = 0.017^∗∗^). The first hypothesis was about the role of education and training in ESE (*t-statistic* = 1.467; *p-value* = 0.143). This hypothesis was accepted at 5% CI for two-tailed. For the third hypothesis, intrinsic motivation significantly predicted the ESE (*t-statistic* = 17.890; *p-value* = 0.00^∗∗∗^). This is the strongest predictor of ESE, while these three independent variables altogether show 62.75% change in ESE. On the other hand, 65.3% change in entrepreneurial behavior is caused by its subsequent predictors. For H_4_, ESE is the biggest predictor of entrepreneurial behavior (*t-statistic* = 35.212; *p-value* = 0.00^∗∗∗^), hence supporting the hypothesis. Moreover, the ESE successfully mediated the role of education and training in entrepreneurial behavior (*t-statistic* = 2.378; *p-value* = 0.00^∗∗∗^); and intrinsic motivation and entrepreneurial behavior (*t-statistic* = 13.375; *p-value* = 0.00^∗∗∗^); hence, supporting H_5_ and H_6_.

**TABLE 5:** Results for structural model.

**Paths**	**H**	**O**	**M**	**SD**	**T-Stats**	***P*-value**	**R^2^**	**Results**
E&T → ESE	H_1_	0.127	0.130	0.053	2.387	0.017**	0.627	*Supported*
Mentorship → ESE	H_2_	–0.067	–0.066	0.045	1.467	0.143		Not supported
IM → ESE	H_3_	0.732	0.731	0.041	17.890	0.000***		*Supported*
ESE →EB	H_4_	0.808	0.810	0.023	35.212	0.000***	0.653	*Supported*
E&T →ESE → EB	H_5_	0.103	0.105	0.043	2.378	0.018**		*Supported*
IM → ESE → EB	H_6_	0.591	0.593	0.044	13.375	0.000***		*Supported*

Significance level *** = 0.005%; ** = 0.05%; H, hypothesis; O, original sample; M, sample mean; E&T, education and training; ESE, entrepreneurial Self-efficacy; IM, intrinsic motivation; EB, entrepreneurial behavior.

## Discussion

This research is based on several hypotheses to analyze the role of education and mentorship in the entrepreneurial behavior of farmers having the mediating risk of ESE. Similarly, the other main relationship of the study was to find the role of education and training, mentorship, and intrinsic motivation in ESE. Of the two major approaches for conducting the research, structural equation modeling was carried out using Smart PLS. A theoretical framework was designed, and questionnaires were sent to the participants. The results supported the hypotheses. The results were also following many researchers, and some were of a different opinion. The possible reasoning for the obtained results is also discussed in this study. A 80% of the respondents were men and 20% were women. They all had different education levels ranging from higher secondary to doctorate.

The cutoff values for reliability are said to be 0.7 (Chang and Chu, 2006). All the values in this study are above 0.70, ranging from 0.852 to 0.934 for alpha (α) reliability and 0.891 to 0.953 for composite reliability. Hence, the data in this study are reliable. The maximum threshold value stated in the literature for factor loadings is 0.6 (Hair et al., 2017; Haq et al., 2020). All the values in this study are above 0.60 except item M3, which is 0.50, acceptable (Peterson and Kim, 2013). The possible reason for getting these results was the authenticity and reliability of the data collected from the participants. Discriminant validity was also tested and found satisfactory for the research. This is also due to the authenticity of the data. For the other criterion, that is, HTMT ratio, the researchers agree that the value should not exceed 0.9, that is, all values should be less (Hair et al., 2017). The results for this study meet this criterion hence, making the data valid for use. In the third phase of data analysis, the data was analyzed for structural model or path analysis using bootstrapping with Smart PLS 3.3.3.

This is usually the subsequent stage of the measurement model. The significance of the relationships is usually expressed in the form of path analysis, which either shows the direct effects or the indirect effects. The direct effects are the general linear regression; however, indirect effects indicate the mediating variables. For the first hypothesis, the role of education and training was analyzed in ESE. This hypothesis was accepted at 5% CI. This is because educating the farmers along with training provided the farmers the opportunity of self-efficacy toward entrepreneurship. Many past researchers have shown similar results in their findings (Karimi, 2019; Wardana et al., 2020).

## Conclusion

The farming industry is flourishing with new technologies and turning toward organic farming with the increase in population. With more demand in organic farming, it is becoming the center of attention for many researchers. This study has also been an attempt to investigate the behavioral and psychological preferences of the farmers. So, the environments and returns for the hard work of farmers could be paid back.

For the third hypothesis, intrinsic motivation significantly predicted ESE. This is the strongest predictor of ESE, while these three independent variables altogether showed a 62.75% change in ESE. On the other hand, 65.3% change in entrepreneurial behavior is caused by its subsequent predictors (Miraglia et al., 2017; Fischer et al., 2019). For H_4_, ESE is the biggest predictor of entrepreneurial behavior, hence supporting the hypothesis. The possible reason behind the acceptance of this hypothesis lies in self-efficacy, as self-efficacy allows the farmers to boost their entrepreneurial behavior (Abraham, 2020).

Moreover, the ESE successfully mediated the role of education and training in entrepreneurial behavior and intrinsic motivation and entrepreneurial behavior; hence, supporting H_5_ and H_6_. This also proved the significance of ESE as a mediator. The possible logic behind its significance is the variable itself. It provides the farmers a satisfaction of dependence on their own, which is necessary for adapting the innovation (Shaheen and AL-Haddad, 2018; Nurlaela et al., 2020). All hypotheses were supported in this study except for mentorship that could not find any significance in predicting ESE. This happened because mentors are not directly involved in mentoring the self-efficacy of the farmers. This study has found certain behavioral preferences of the farmers like any other professionals regarding their ESE. Mentorship did not find to have any role to play in predicting ESE. However, education training and intrinsic motivation are major driving forces for ESE and entrepreneurial behavior. The current study is a major contribution in psychology concerning farmers who have not been investigated previously taking their behaviors into account. This research has several implications for the future researchers and e-commerce players who are interested in repeating this research with their available resources in different regions. These can be exploited well in finding new avenues for certain researches like this.

## Data Availability Statement

The original contributions presented in the study are included in the article/supplementary material, further inquiries can be directed to the corresponding author.

## Ethics Statement

The studies involving human participants were reviewed and approved by Zhejiang University of Technology, China. The patients/participants provided their written informed consent to participate in this study. The study was conducted according to the Declaration of Helsinki.

## Author Contributions

BH and JW conceived and designed the concept. QZ, ZT, and JZ collected the data and provided technical support. SW helped in resources and validation. BH wrote the manuscript. YL contributed to draft manuscript preparation. All authors read and agreed to the published version of the manuscript.

## Conflict of Interest

The authors declare that the research was conducted in the absence of any commercial or financial relationships that could be construed as a potential conflict of interest.

## Publisher’s Note

All claims expressed in this article are solely those of the authors and do not necessarily represent those of their affiliated organizations, or those of the publisher, the editors and the reviewers. Any product that may be evaluated in this article, or claim that may be made by its manufacturer, is not guaranteed or endorsed by the publisher.
